# Increasing access without compromising quality: optimizing telemedicine care in pediatric rheumatology

**DOI:** 10.3389/fped.2025.1700036

**Published:** 2025-11-19

**Authors:** S. Jones, M. Toth, A. B. Liu, E. M. Morgan, J. Leal, Y. I. Goh, K. Hayward

**Affiliations:** 1 Department of Pediatrics, Texas Children’s Hospital and Baylor College of Medicine, Houston, TX, United States; 2 Department of Pediatrics, Nemours Children’s Health, Orlando, FL, United States; 3 Seattle Children’s Research Institute, Seattle, WA, United States; 4 Department of Pediatrics, Seattle Children’s Hospital & University of Washington School of Medicine, Seattle, WA, United States; 5 Jenny—Parent Partner, PR-COIN, Columbus, OH, United States; 6 Division of Rheumatology, The Hospital for Sick Children, and Child Health Evaluative Sciences, SickKids Research Institute, Toronto, ON, Canada

**Keywords:** telemedicine, juvenile idiopathic arthritis, physical examination, digital health, implementation science, medical education, pediatrics

## Abstract

Juvenile idiopathic arthritis (JIA) is a chronic immune-mediated condition affecting approximately 1 in 1,000 children in the United States. Without appropriate treatment, JIA can lead to permanent disability, chronic pain, and impaired quality of life. Targeted treatment strategies have enabled some patients to achieve sustained inactive disease and improved outcomes. However, the ongoing workforce shortage of pediatric rheumatologists poses a barrier to accessing effective care for many patients and families. Telemedicine is a potential solution to improving access to care for patients who travel significant distances to obtain pediatric rheumatology healthcare. During the COVID-19 pandemic, telemedicine saw rapid adoption with exponential uptake in use during unprecedented circumstances and resulted in inconsistent implementation of this emerging healthcare modality. While virtual visits offer many benefits, they often fail in gathering critical data elements required for measuring and tracking health outcomes, engaging in data-driven shared decision making, population health management and secondary research. In response to these challenges, the Pediatric Rheumatology Care and Outcomes Improvement Network (PR-COIN) Digital Health Workgroup conducted a needs assessment among its members to identify opportunities and barriers for optimizing telemedicine visits for patients with JIA. Based on this assessment, potential interventions and instructional materials were developed and compiled into a “change package” to assist rheumatology teams in planning, implementing and evaluating improvements to telemedicine visits at their centers. This change package is publicly available on the PR-COIN website for adoption and use (https://www.pr-coin.org/center-tools-resources). Continuous evaluation and adaptation of these tools are essential as telemedicine practices continue to evolve.

## 1 Introduction

Juvenile idiopathic arthritis (JIA) is a chronic immune-mediated condition, with an estimated prevalence in the United States (US) of approximately 1 per 1,000 children (1, 2). Without appropriate treatment, the condition can result in permanent disability, chronic pain, and impaired health related quality of life (QOL). While there is currently no cure for JIA, targeted treatment strategies can enable approximately 50% of patients to achieve sustained remission and greatly improved long-term outcomes (3). Despite the availability of modern therapies, the shortage of pediatric rheumatologist specialists has led to delays in timely access to care for many patients and families (4). Compounding the shortage of appointments, the long distance that patients need to travel to access pediatric rheumatology care is one of the biggest barriers. A 2006 study found that patients travelled an average of 60 miles to see their nearest pediatric rheumatologist in the US (5). Telemedicine has emerged as a powerful solution to overcome the barrier of distance in pediatric rheumatology (6). The widespread adoption of telemedicine during the COVID-19 pandemic demonstrated its acceptability among patients, leading to decreased travel times and minimized lost work and school time (7). However, uncertainties persist about the best practices for the utilization of telemedicine in pediatric rheumatology care, given that in-person physical exams have historically been fundamental for disease activity assessments and medical decision-making.

## 2 Background

Prior to the pandemic, telemedicine was used in limited capacity to deliver pediatric rheumatology care to selected patients experiencing geographic barriers to access. In this model, patients travelled to a local healthcare hub within their community and received care from non-specialist healthcare providers in conjunction with a specialist in virtual attendance. Although these community healthcare hubs were not staffed with pediatric rheumatologists, the staff were able to conduct physical examinations and access to the equipment necessary to facilitate telemedicine visits (6, 8). This historic telemedicine practice was limited to healthcare providers who had an interest in serving remote populations through telemedicine.

The use of telemedicine for pediatric rheumatology care expanded rapidly during the COVID-19 pandemic, but this rapid implementation occurred in the absence of established guidelines and best practices (9). Healthcare providers had to quickly adapt and improvise to practice medicine using virtual medium without validated tools, resources, or standardized protocols (10). Consequently, the delivery of pediatric rheumatology care using telemedicine has been inconsistent, relying largely on individual providers' technological proficiency and local institutional support. Moreover, many patients and caregivers were unsure of what to expect from a telemedicine visit or how to prepare for one. Several publications have highlighted barriers and challenges faced by both patients and providers in the initial delivery of pediatric rheumatology care using telemedicine, including difficulties in assessing psychosocial risk, obtaining vital signs, and coordinating ancillary services such as laboratory work, imaging, nursing, and social work (11, 12).

One of the greatest concerns is the inability of providers to perform the gold standard hands-on physical assessment to assess for active joint disease. In attempt to replicate physical exams in a virtual setting, many pediatric rheumatology providers adapted an existing screening technique called the pediatric Gait, Arms, Legs and Spine (pGALS) assessment for use over telemedicine, in a modified video-pGALS (V-pGALS) (10, 13). While a small study demonstrated the validity of the V-pGALS, it remains unclear whether the results obtained using the V-pGALS in the virtual setting are comparable to the active joint counts historically obtained during in-person assessments (14). The lack of established validity for virtual physical exams has broad implications for both individual patient care as well as population-based care and research.

Pediatric Rheumatology Care and Outcomes Improvement Network (PR-COIN) is a multi-center learning collaborative that spans 23 pediatric centers across the United States and Canada and comprised of over 300 members, including healthcare providers, research personnel, clinical staff, and patients, families, and caregivers^1^. The cornerstone of PR-COIN is the use of robust quality improvement methodology to standardize, study and improve the care of patients with JIA across North America. PR-COIN utilizes a Treat-to-Target (T2T) shared decision-making approach to JIA treatment decisions. This approach has been demonstrated to improve outcomes in multiple chronic conditions, is widely accepted as the standard of care in adult rheumatoid arthritis (RA) treatment, is supported by international consensus recommendations in pediatric rheumatology, and is backed by evidence for use in JIA specifically (15–17). A key element of the T2T approach is having an appropriate outcome measure to track disease activity.

PR-COIN has identified a core group of data elements to be collected at every patient visit to track JIA disease outcomes in part to facilitate a T2T strategy to improve rates of inactive disease, and in part to enable tracking of a broader set of quality measures (18). These Critical Data Elements (CDE) include components required to calculate the clinical Juvenile Arthritis Disease activity score (cJADAS), (i.e., complete joint count, physician global assessment of disease activity, patient assessment of overall well-being) as well as components of the American College of Rheumatology provisional criteria for inactive disease, and Patient Related Outcomes (PROs) such as arthritis related pain (19–21). While these CDEs are items that can, in theory, be gathered during telemedicine encounters, data on the quality of pediatric rheumatology virtual visits early in the pandemic showed that collection rates for CDE in virtual visits were significantly lower than for in-person care (22, 23).

Recognizing the challenges being faced by pediatric rheumatology healthcare providers and their patients/caregivers related to the abrupt adoption of telemedicine, PR-COIN established a Digital Health Workgroup. The mission of this workgroup is to establish, implement, and promote quality telemedicine care and digital care models to connect children and adolescents with rheumatic disease with their healthcare providers unbound by their physical location. One of the priorities identified by the PR-COIN Digital Health Workgroup was the need to help patients and healthcare providers prepare for their telemedicine visit. This project aimed to develop a change package of interventions designed to help pediatric rheumatology healthcare providers optimize and standardize telemedicine care for JIA patients and to help patients and caregivers prepare for and understand what to expect during a telemedicine visit.

## 3 Needs assessment

The Digital Health Workgroup of PR-COIN began with a multi-modal needs assessment to identify gaps in knowledge, skills and resources related to conducting pediatric rheumatology telemedicine visits. The assessment was informed by a literature search of PubMed for published articles on telemedicine use in pediatric rheumatology. Currently, literature in the public domain largely consists of review articles, description of telemedicine usage during the COVID pandemic and reports of patient and provider satisfaction with telemedicine encounters. Several articles also identified challenges associated with telemedicine visits for patients with JIA, including: obstacles in conducting thorough physical examinations and discrepancy in collection of data elements between virtual and in person visits (12, 22, 23). There was no published data identified with clinical outcomes for JIA patients seen by telemedicine. Based on this literature review, a survey of both quantitative and qualitative elements around current post-pandemic telemedicine practices was constructed. Questions included: Site location, volume of telemedicine visits for JIA patients, methods for collection of PROs for both in person and telemedicine visits, details around staff preparation for visits, use of standardized methods for virtual physical exams, as well as open ended questions around barriers to improving standard collection of CDE in telemedicine visits. Content validity was established through expert review from members of the PR-COIN telemedicine work group who evaluated each item for relevance and clarity. An email with a link to the finalized REDCap survey link was sent to the PI's for all 23 active sites PR-COIN network Responses were received from 91% (21/23) of network sites. Results indicated that 33% (7/21) of sites were still utilizing telemedicine for 10%-25% of patient visits. Only 57% (12/21) of responding sites regularly collected PROs during telemedicine visits; 33% (7/21) sites collected PROs electronically, 14% (3/12) collected them verbally, and 10% (2/21) used both methods. The remaining sites either lacked routine methods for PRO collection during virtual visits or noted that it was provider dependent. In comparison, for in-person visits, 95% (20/21) of the responding sites had systems in place to collect PROs, [33% (7/21) electronically, 52% (11/21) paper and 14% (3/21) both]. Regarding physical examination approaches during telemedicine visits, there was little standardization, with only 10% (2/21) of sites indicating the use of a standard virtual exam. Seventy-one percent (125/21) of sites reported some provider use of V-pGALS or another adaptation of pGALS. Seventy-one percent (15/21) of sites had staff coordination post-visit, primarily for scheduling follow-up visits. Barriers to effective telemedicine visits identified by PR-COIN sites included items such as: patient in clothing that covered their joints, locations that prevented adequate virtual joint exam, lack of systems for virtual PRO collection, and concerns about reliability of active joint counts identified by virtual physical exams. Respondents also suggested the need for educational materials, automated/electronic methods for PRO collection and other CDE collection via the medical record, and standardized templates.

## 4 Materials development

**Figure 1:**
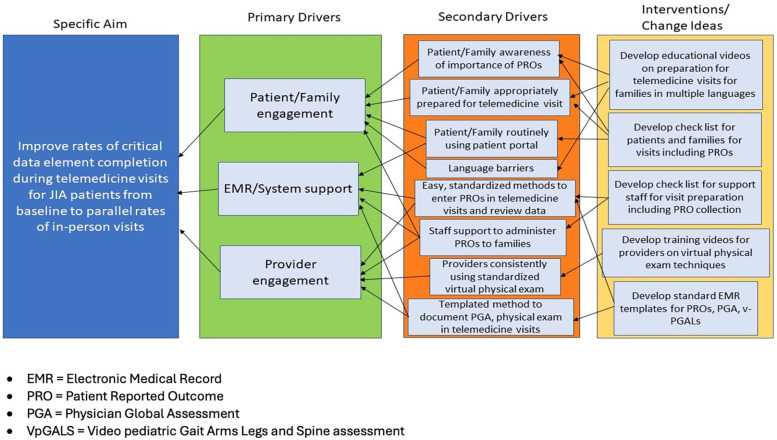
Key driver diagram.

Based on the needs assessment and site survey, a Key Driver Diagram was created as a framework for developing and adopting proposed interventions to improve collection of CDE. (Figure 1: KDD). Site survey participants free test responses to the question, “What do you see as your sites biggest barriers to improving standard collection of patient reported outcomes in telemed visits and virtual physical exams” were analyzed qualitatively using an inductive thematic approach. One author (KH) coded the responses and the themes were reviewed and approved by co authors (MBT, EM, GY) Three themes emerged: inadequate preparation of patients, incomplete or inconsistent systems processes and inadequate provider engagement with telemedicine as a modality. These thematic groupings were used to generate three primary drivers for improvement in the KDD: patient and family engagement, electronic medical record (EMR) and system support, and provider engagement. Corresponding interventions were developed to support patients, staff and clinicians in navigating telemedicine visits. The intervention materials were assembled as part of a broader change package in order to promote the implementation of best practices for optimizing telemedicine care for families with JIA. The change package includes an instructional guide, suggested interventions, and quality improvement tools to facilitate and monitor progress.

To address concerns around family preparedness for telemedicine visits, resources were developed, including a pre-visit video for patients and caregivers. This video is entitled “Telemedicine Visit: How to Prepare for your JIA Visit (YouTube)” and offers tips on optimal clothing to facilitate joint examination, suitable space for movement during the exam, and tips for checking internet bandwidth prior to visits. Additional resources developed include patient facing handouts with a checklist for pre-visit preparation and information on the importance of PRO collection titled, “What are PROs (Patient Reported Outcomes) and Why Do They Matter?”

Resources for staff and systems include three checklists covering scheduling telemedicine visits, rooming patients during their telemedicine visit, and conducting post-visit wrap-up, emphasizing opportunities for CDE collection based on site-specific processes.

Interventions for provider engagement in telemedicine visits focused on the virtual physical exam. A standardized approach to the physical examination using the V-pGALS was identified as the best practice to date (14). The change package includes a guide to performing V-pGALS with a video demonstration and a tip sheet for teaching the video exam technique. Additional provider-facing resources include guides for patient selection for telemedicine visits and sample electronic medical record templates to modify for their practices.

**Figure 2:**
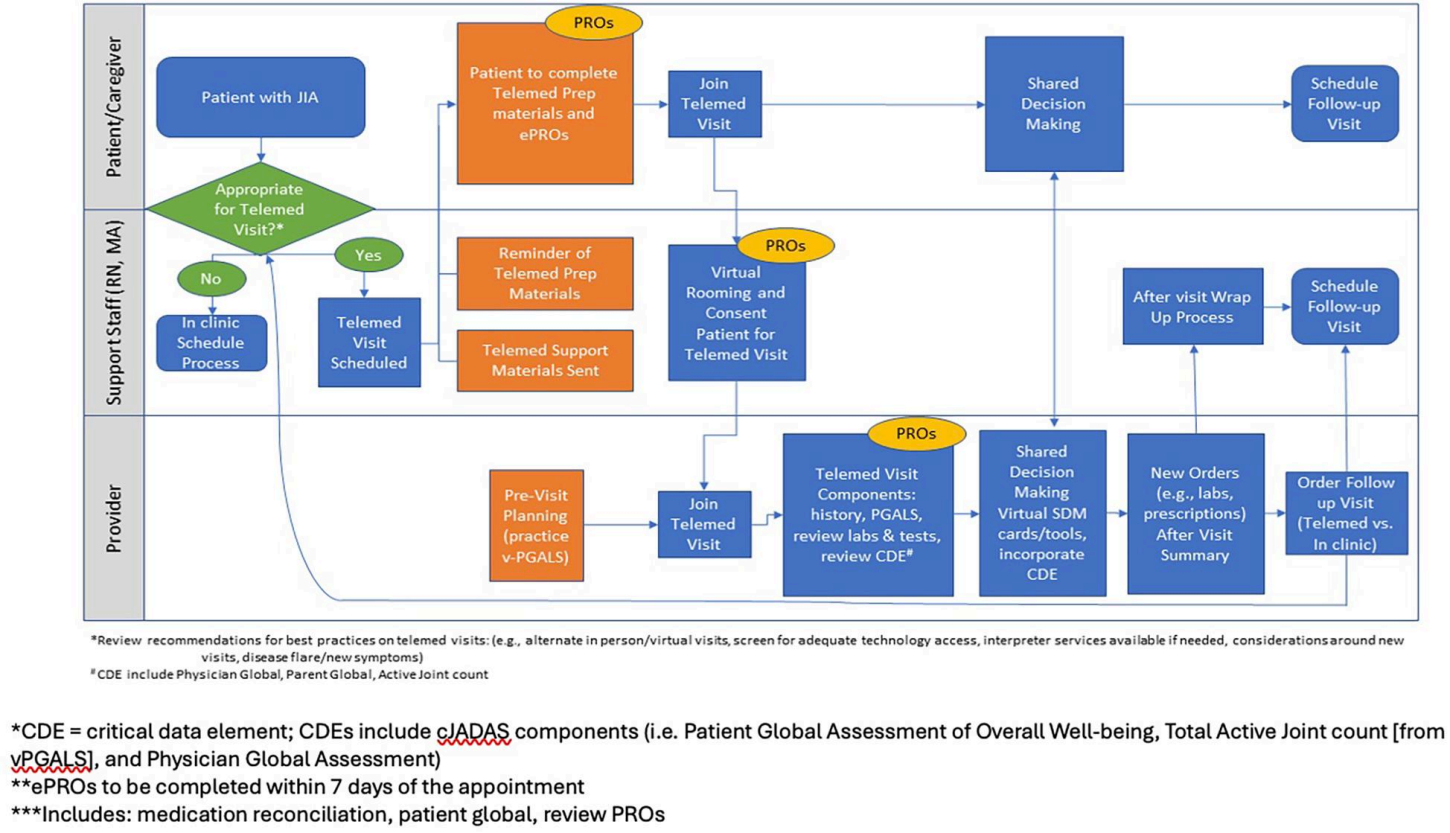
Process map.

The change package also provides a roadmap for planning and implementing change ideas, from identifying an implementation framework to identifying interested parties and defining the role of telemedicine within their organization, identifying the ideal patients to see using telemedicine, creating a site-specific process map, establishing evaluation methods, choosing and implementing specific interventions, and tracking outcomes. An example process map with suggested practice changes for patients/caregivers, support staff, and healthcare providers is included in the change package Figure 2.

### 4.1 Incorporating feedback and dissemination

PR-COIN hosts biannual two-day learning sessions occurring in the spring and the fall. The workgroup utilized the learning sessions to present and collect feedback on the components of the change package from participants of the learning sessions which includes pediatric rheumatologists, advanced practice providers and pediatric rheumatology researchers from across the PR-COIN North American network. Participants from the Spring 2024 Learning Session on April 25 and 26th, 2024 included 86 unique attendees representing 19 of 22 participating PR-COIN centers. Attendees were provided with a draft of the change package to review as part of the learning session with a survey asking for free-form comments about the change package and the VpGALS video and tip sheet (2 respondents). Additional feedback was solicited during a live session presentation of the video by author (KH). Feedback from the live session and survey were compiled. Actionable suggestions included shortening the length of the video and adding in reference diagrams. Minor additions were then made to the Change Package, which was presented in its final version at the PR-COIN Fall 2024 Learning Session on September 26 and 27, 2024 in Seattle, Washington to 91 unique attendees from 21 of the 23 participating centers by authors (KH and MT). The final Change Package was published on the PR-COIN internal network platform as well as on the PR-COIN public-facing website.^2^

## 5 Discussion

As we transition into the post-pandemic era, it is clear that telemedicine has emerged as a powerful option for healthcare delivery and an alternative to in-person visits (24). Comparison of research prior to vs. during the COVID-19 pandemic indicates improvement in patient and healthcare provider satisfaction with telemedicine use (6, 7, 25, 26). This trend is expected to persist as both the public and healthcare providers grow more accustomed to this method of accessing care. However, the optimal role of telemedicine visits in rheumatology, particularly regarding its impact on outcomes for patients with juvenile idiopathic arthritis (JIA), remains to be fully elucidated (27–29).

Our recent needs assessment has highlighted that CDEs necessary for tracking disease activity and improving outcomes in JIA patients are still not being routinely collected during telemedicine visits (30). The inconsistent collection of these CDEs not only compromises the quality of individual patient care, but also impedes efforts to enhance population health over time (18). CDEs and patient outcomes are integral to both quality improvement and clinical research; an increase in missing data due to virtual visits dilutes our capacity to learn and advance as a field.

Among the frequently missing data points during telemedicine visits are total active joint counts and physician global assessments, which historically relied on in-person examinations (30). Although the V-pGALS approach—a standardized telemedicine physical exam—has gained traction across our network, many clinicians still feel uneasy about assigning a numerical active joint count without a direct examination of the patient. Qualitative feedback gathered from PR-COIN learning session indicated that different providers have addressed this uncertainty in various ways: some leave the field blank, while others assign what they consider an “estimated” active joint counts based on virtual assessments. While this latter approach may reduce missing data, it could also introduce additional uncertainty in data collection over time, especially if the accuracy of virtual visits is inferior to that of in-person evaluations. Until formal validation studies are conducted, we advocate for consistent reporting of active joint counts during all visits, even if they are estimates. This approach acknowledges that even in-person joint examinations have variable interrater reliability and may not fully detect active arthritis (31). Consistent data collection over time, regardless of the visit format, will enable us to assess the validity of telemedicine examinations and their impact on care outcomes.

As the pediatric rheumatology community grows more comfortable with virtual care as an accepted aspect of practice, there arises an urgent need to develop and incorporate explicit training in telemedicine practices for learners in our field (32). Currently, very few curricula exist to formally train residents and fellows in conducting telemedicine visits (33). The American College of Rheumatology (ACR) has recently published competencies for telehealth visits, providing a foundational set of 24 skills expected for physicians engaging in virtual patient encounters (34). Based on these competencies, emerging curricula in adult rheumatology are being developed to help fellows acquire the specific competencies necessary for telemedicine visits (35). Notably, a virtual rheumatology objective structured clinical examination was created, implemented and evaluated for trainees in adult rheumatology (36) These curricula emphasize strategies for building rapport over telehealth platforms and optimizing physical examination components suitable for virtual settings. However, existing training curricula are focused on adult rheumatology practice and do not address the challenges of conducting virtual joint examinations in young patients, who may have limited attention spans and may not be able to follow instructions as effectively on their own. Our hope is that the V-pGAL's demonstration video and tips sheets for providers can be utilized to develop more robust curricular resources for training pediatric rheumatology providers in virtual visits.

In addition to ensuring adequate education for our provider trainees, it is crucial that healthcare systems learn and evolve to adequately support telemedicine visits. The electronic collection of PROs presents an appealing opportunity for integrating reliable assessments of patient perceptions into care. Despite the availability of these platforms, we observed that only a minority of centers within the PR-COIN network currently utilize these technologies. Healthcare systems need to establish reliable methods for obtaining PROs during telehealth visits that are comparable to those used in in-person consultations.

Our goal was to provide a comprehensive set of interventions to assist pediatric rheumatology centers in implementing strategies to enhance the quality of telemedicine visits. However, we encountered barriers related to the variability of EMR systems across different institutions. Even when institutions use the same EMR software, differing configurations and user interfaces complicated the provision of easily applicable solutions for practitioners. Additionally, discrepancies in institutional policies regarding communication with patients through EMR interfaces, such as secure patient portals, further complicated efforts to disseminate widely applicable tools. For instance, we noted that some providers' institutions required branded videos or had previously developed telehealth information videos that, while potentially useful, did not specifically address the unique needs of rheumatology visits.

To our knowledge, this is the first change package created to optimize the delivery of care for JIA patients via telemedicine. There are several limitations to this work in its current form. Currently we do not have outcomes data to assess the validity of the proposed interventions. More work will be required to determine if implementation of elements of the change package result in improvement of collection of CDE during telemedicine visits and ultimately lead to improved care for JIA patients. The change package is currently available only in English and Spanish. With advancements in artificial intelligence, it may allow translation to be easier and cost-effective. However, given that the change package was developed in a North American context, it will be crucial to review the content prior to implementation in other countries to ensure cultural relevance. We created this change packet based on constructs and resources available across the PR-COIN network; those aiming to implement in other settings should select an implementation framework that aligns with their practice environment and may need to modify components to fit their specific context.

The creation of this change package illustrates what can be accomplished through gathering end-user feedback and employing continuous feedback mechanisms to refine an acceptable solution that can be adapted for practical use. While this change package has been established as a current snapshot of best practices, adherence to specific interventions may vary, and they may not always be implemented as intended given the rapidly evolving nature of telemedicine and EMR formats, including anticipated changes related to incorporation of artificial intelligence. Additional post-implementation testing and refinement of the change package will be necessary to continually enhance best practices as the application of telemedicine evolves over time.

## 6 Permission to reuse and copyright

Permission to use and reference the V-pGALS was obtained from Paediatric Muscoloskeletal Matters (37).

The PR-COIN telemedicine change package is licensed under CC BY-NC 4.0. Use or adaptation reproduction of the PR-COIN telemedicine change package, with the exception of the Clinic Checklist, must provide attribution to PR-COIN. Note that only the indicated sections of the Clinic Checklist may be modified; please see the Clinic Checklist for additional copyright information.

## Data Availability

The original contributions presented in the study are included in the article/Supplementary Material, further inquiries can be directed to the corresponding author.

## Footnotes

1 Available online at: https://www.pr-coin.org

2 Available online at: https://www.pr-coin.org/center-tools-resources

## Group member of the pediatric rheumatology care and outcomes improvement network

We are grateful for additional contributors to the Telemedicine Improvement Project:

Anabelle Burgos, BS, RN (Nemours Orlando—Orlando, FL). Brian Feldman, MD, MSc (The Hospital for Sick Children—Toronto, Ontario, Canada). Erik Friedrichsen (Seattle Children's Research Institute, Seattle, WA). Helen Foster (Newcastle University—Newcastle Upon Tyne, United Kingdom). Nidhi Naik, MPH (Seattle Children's Research Institute—Seattle, WA). Jade Singleton, PhD, MPH (Seattle Children's Research Institute—Seattle, WA). Katie Young (PR-COIN Parent Advocate).
